# Improving Thermal Conductivity Coefficient in Oriented Strand Lumber (OSL) Using Sepiolite

**DOI:** 10.3390/nano10040599

**Published:** 2020-03-25

**Authors:** Hamid R. Taghiyari, Abolfazl Soltani, Ayoub Esmailpour, Vahid Hassani, Hamed Gholipour, Antonios N. Papadopoulos

**Affiliations:** 1 Wood Science and Technology Department, Faculty of Materials Engineering & New Technologies, Shahid Rajaee Teacher Training University, Tehran 1678815811, Iran; vahid.hassani@gmail.com; 2 Faculty of Civil Engineering, Shahid Rajaee Teacher Training University, Tehran 1678815811, Iran; abolfazl_soltani2003@yahoo.com; 3 Department of Physics, Faculty of Sciences, Shahid Rajaee Teacher Training University, Tehran 1678815811, Iran; esmailpour@sru.ac.ir; 4 Department of Mechanical Engineering, Shahid Rajaee Teacher Training University, Tehran 1678815811, Iran; gholipour@sru.ac.ir; 5 Laboratory of Wood Chemistry and Technology, Department of Forestry and Natural Environment, International Hellenic University, GR-661 00 Drama, Greece

**Keywords:** composite panels, mineral materials, oriented strand lumber (OSL), sepiolite, thermal conductivity coefficient

## Abstract

An issue in engineered wood products, like oriented strand lumber (OSL), is the low thermal conductivity coefficient of raw material, preventing the fast transfer of heat into the core of composite mats. The aim of this paper is to investigate the effect of sepiolite at nanoscale with aspect ratio of 1:15, in mixture with urea-formaldehyde resin (UF), and its effect on thermal conductivity coefficient of the final panel. Sepiolite was mixed with UF resin for 20 min prior to being sprayed onto wood strips in a rotary drum. Ten percent of sepiolite was mixed with the resin, based on the dry weight of UF resin. OSL panels with two resin contents, namely 8% and 10%, were manufactured. Temperature was measured at the core section of the mat at 5-second intervals, using a digital thermometer. The thermal conductivity coefficient of OSL specimens was calculated based on Fourier’s Law for heat conduction. With regard to the fact that an improved thermal conductivity would ultimately be translated into a more effective polymerization of the resin, hardness of the panel was measured, at different depths of penetration of the Janka ball, to find out how the improved conductivity affected the hardness of the produced composite panels. The measurement of core temperature in OSL panels revealed that sepiolite-treated panels with 10% resin content had a higher core temperature in comparison to the ones containing 8% resin. Furthermore, it was revealed that the addition of sepiolite increased thermal conductivity in OSL panels made with 8% and 10% resin contents, by 36% and 40%, respectively. The addition of sepiolite significantly increased hardness values in all penetration depths. Hardness increased as sepiolite content increased. Considering the fact that the amount of sepiolite content was very low, and therefore it could not physically impact hardness increase, the significant increase in hardness values was attributed to the improvement in the thermal conductivity of panels and subsequent, more complete, curing of resin.

## 1. Introduction

Oriented strand lumber (OSL) is a widely studied engineered wood to satisfy the demand for structural wood products [1,2,3]. A wide variety of wood species have been applied to produce OSB and OSL panels with a high quality, including pine, spruce, rubberwood, and poplar [2,4,5]. One constant problem in engineered wood and wood-composite manufacturing factories is the low thermal conductivity coefficient of wood, preventing the fast transfer of heat into the core of composite mats [6,7,8]. A lengthy heat transfer to the inner parts prolongs the resin-curing process, which is vital for a fast and effective production line. Increasing the water content of composite mats cannot be considered practical due to the consequent problems, like the occurrence of blows in panels. 

Nanotechnology has been reported to improve the properties and eliminate some drawbacks in many composites and materials [9,10,11,12,13], although changes can occur in the material properties when the size range is reduced to nanometer scale [14]. For wood and wood-composites, different nanomaterials were utilized to further improve fire retardancy, biological resistance, water repellency, and mechanical properties [15,16,17,18,19,20,21,22,23]. Recently, nanowollastonite was applied with urea formaldehyde resin, which improved the thermal conductivity coefficient and, consequently, the mechanical and physical properties of OSL [12]. It was reported that that the fortification of UF resin with 10% nanowollastonite was considered as an optimum level. The mechanism involved in the fortification of UF resin with nanowollastonite was attributed mainly to the following: (i) nanowollastonite compounds made active bonds with the cellulose hydroxyl groups, putting them out of reach for the water molecules to make bonds with and (ii) the high thermal conductivity coefficient of wollastonite improved the transfer of heat to different layers of the OSL mat, facilitating better and more complete resin curing.

The approach of the present work is to look at ways of improving the thermal conductivity coefficient of OSL. Particularly, the scope of this study is to investigate the effect of using sepiolite at nanoscale, in a mixture with urea-formaldehyde resin (UF), and to study its effect on the thermal conductivity coefficient of the final panel. Sepiolite was selected because its fibrous-like structure is believed to be more easily dispersed in the polymer matrix compared to platelet-like minerals, like montmorillonites (MMT) [24]. Furthermore, sepiolite may prevent flocculation if it is distributed within the network matrix of the polymer and may also reduce filler agglomeration [25]. With regard to the fact that an improved thermal conductivity would ultimately be translated into a more effective polymerization of the resin, the hardness of the panel is measured to find out how the improved conductivity affects the hardening of the resin. 

Sepiolite is a common hydrated magnesium silicate with half unite-cell formula Mg_4_Si_6_O_15_(OH)_2_.6H_2_O. The structure of sepiolite is composed of two bands of silica tetrahedrons linked by magnesium ions in octahedral coordination, and the silica tetrahedrons extend as a continuous layer with an inversion of the apical ends every six units [26,27,28]. Sepiolite has nanometer tunnel structure, which shows a micro-fibrous morphology with a particle size in the 2–10 µm length range [29], while its specific surface area has a value close to 320 m^2^/g [30]. Sepiolite is mainly used as a reinforcement to enhance polymer properties because of its large surface area [31,32,33,34], while studies using sepiolite as a filler on formaldehyde-based resins are limited. In a recent study, various proportions of sepiolite were applied to substitute wheat flour as a resin filler, in the plywood manufacture [35]. The results revealed that the sepiolite with the cured resin made a tough surface which resulted in a maximum improvement in wet shear strength by 31.4%. After an exhaustive search in the related literature, we were not able to find a study in which sepiolite is applied in a mixture with UF resin directly to wood raw material. Therefore, the aim of this paper is to investigate the effect of sepiolite at nanoscale with an aspect ratio of 1:15, in a mixture with urea-formaldehyde resin (UF), and its potential effect on the thermal conductivity coefficient of the final panel.

## 2. Materials and Methods

### 2.1. Nano-Sepiolite Application 

Sepiolite stone was taken from Tanbo village located in the Senderk region, south of Minab city (Minab, Iran). In this region, sepiolite rocks were cropped out in the most western part of Makran Ophiolite zone where it joined Zagros Fold Belt. The results of XRF analysis demonstrated the chemical composition of the sepiolite (Table 1). The XRF analyses were performed by Philips PW1410 apparatus (CAE, Austin, TX, USA). The X-ray fluorescence analyzer used for this study was available from Beam Gostar Taban Laboratories (BGTL, Tehran, Iran). The identified fibrous minerals occurred within the Miocene harzborgite conglomerate. The length of macro-fibers derived from sepiolite stone reached 60 cm and the slender ratio (length to width) was more than 1200 [36]. The loss on ignition (L.O.I.) after 10 h heating at 900 °C was 18.88 %. It is hypothesized that the fibrous shape with a rather high aspect ratio can act more efficiently as a reinforcing filler in resin. However, a comparison study focused on only the different aspect and slender ratios of a material should be carried out to conclude this point. 

**Figure 1:**
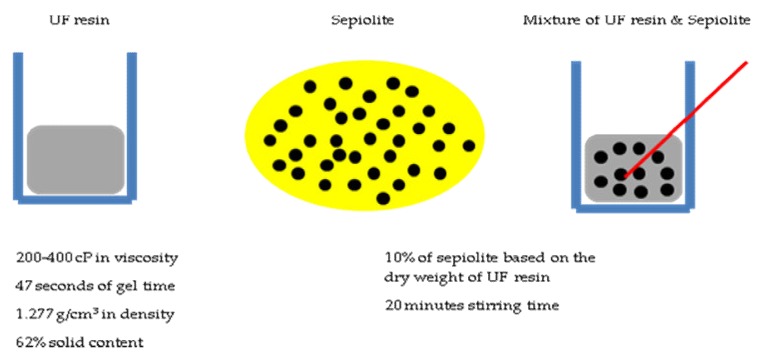
Flow diagram of resin–sepiolite mixture preparation.

Sepiolite was mixed with UF resin for 20 min, using a magnetic stirrer, to make sure that sepiolite nanostrands were evenly mixed with the resin. Once resin and nano-sepiolite were mixed, the mixture was sprayed onto wood strips in a rotary drum. Ten percent of sepiolite was mixed with the resin, based on the dry weight of the UF resin. The flow diagram of this experimental design is presented in Figure 1.

### 2.2. SEM Imaging

Scanning Electron Microscope (SEM) imaging was carried out at Beam Gostar Taban Laboratories, Tehran. A completely new field emission scanning electron microscope (FE-SEM), TESCAN-MIRA III model was used for imaging, made in Czech Republic. Samples were first gold-coated for a thickness of 10 nm, before FE-SEM imaging. 

### 2.3. Temperature Measurement at the Core Section of the Mat 

**Figure 2:**
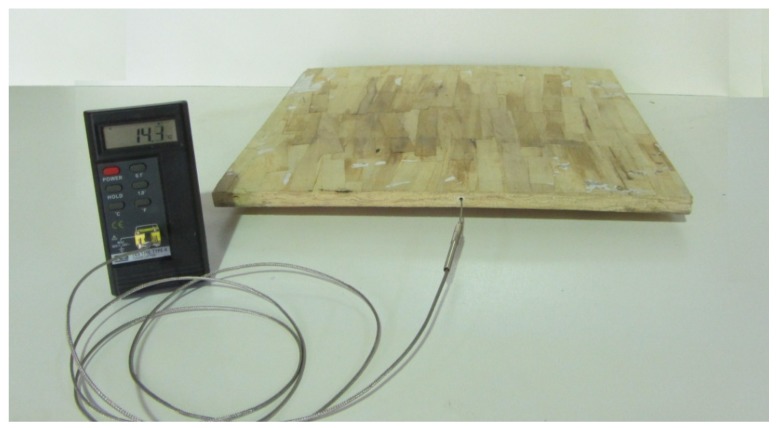
Measurement of temperature at the core of a mat during hot-press using a digital thermometer with 0.1 °C precision; the sensor probe is inserted into the core of the oriented-strand lumber mat.

The temperature at the core section of the mat was measured, with 0.1 °C precision, with a digital thermometer with a temperature sensor probe at 5 s intervals (Figure 2). The probe was inserted, in the horizontal direction, for approximately 50 mm into the core of the mat. Temperature measurement was begun immediately after the hot plates reached the stop-bars. This procedure was also applied by other researchers [37,38,39].

### 2.4. Thermal Conductivity Measurement 

**Figure 3:**
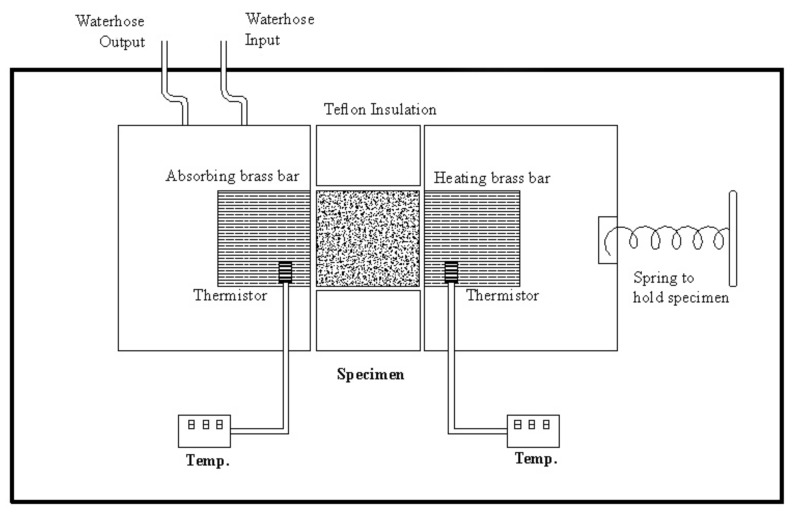
Schematic representation of the apparatus used for the measurement of thermal conductivity [40].

Thermal conductivity coefficient was calculated based on Fourier’s Law for heat conduction, using an apparatus by Iranian Precise System Co. (IPS) (Figure 3). Cylindrical samples were cut with 30 mm in diameter and 16 mm in length; in order to achieve better insulation, the circumferential area of the samples was covered with silicone adhesive. The thermal conductivity of wood and wood composites is strongly influenced by their moisture content. Therefore, all specimens were conditioned (25 ± 2 °C, and 65% ± 3% relative humidity) for a month to reach to a final moisture content of 7.5% at the time of measurement. Samples were radially positioned in an annular Teflon holder to minimize the radial heat conduction, and afterwards were axially placed between the heating and absorbing brass rods, which themselves were also radially covered by Teflon insulations; the whole system was enclosed inside a thick layer of glass wool to ensure that the heat conduction is axial and one-dimensional within engineering accuracy. The heating brass bar heated the specimen from one side at 130 °C (Figure 3), while the other face of the cylindrical sample was touched by the absorbing brass bar connected to a heat sink. Temperature of the absorbing brass bar was 30 ± 2 °C during the test for all specimens. Each specimen was left in the system for an approximate time of half an hour until all the thermistors read a constant temperature, which meant the heat flux was truly constant and at steady state, and consequently could be well represented by Fourier’s Law (Equation (1)). In order to measure the rate of heat-transferring, temperatures at the two ends of each sample were read and registered. Thermal conductivity was then calculated using Equation (2)

(1) $$Q=k\;A\;\;\frac{T_{1}-\;T_{2}}{\Delta {\;}_{x}}$$

(2) $$k=\;\frac{\;\;Q\;\times \;L\;\;}{A\;\times \Delta T}$$

where: k = coefficient of thermal conductivity (W·m^−1^·K^−1^); Q = heat flux (W); A = cross-section area of specimens (m^2^); ΔT = temperature difference (T1–T2) (K); Δ*x* = thickness of specimens (m); L = specimen thickness (m).

### 2.5. Specimen Preparation 

**Figure 4:**
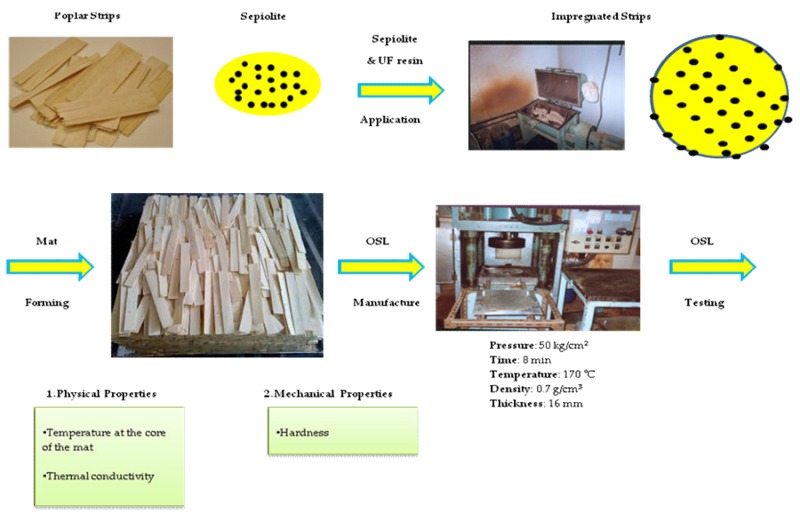
Flow diagram of the experimental procedure.

Poplar wood with a density of 0.42 g/cm^3^ was used in the present study. Logs were peeled and dried to a moisture content of 6%, before stripping the veneers. The strips were 150 × 20 × 1 mm in dimension; their length was aligned in the longitudinal direction of the logs. Strips were dried for 48 h at 50 °C and then kept in plastic bags. Urea–formaldehyde resin (UF), with 200–400 m Pa s in viscosity, 47 s of gel time, and 1.277 g/cm^3^ in density, was purchased from Iran Choob Co. (Ghazvin, Iran); the UF resin contained 62% solids. As a hardener, 2% ammonium chloride was added to resin. Ammonium chloride was purchased from Galaxy Chemistry Co. (Tehran, Iran). OSL panels with two resin contents, namely 8% and 10% were manufactured. The mixture of resin and hardener was sprayed on the strips in a rotary drum. Afterwards, the strips were manually arranged in a forming to be hot-pressed for 8 min at 170 °C. The final thickness of panels was 16 mm and target panel density was 0.7 g/cm^3^. Five replicates were produced for each of the four treatments; totally, 20 panels were produced. The produced panels were edge-trimmed to produce a 450 × 450 mm^2^ final panel dimension. After the hot-press, the produced panels were conditioned (25 ± 2 °C, and 65 ± 3% relative humidity) for a month, before being cut and tested. The flow diagram of this experimental procedure is presented in Figure 4.

### 2.6. Hardness Measurement

Hardness tests were done using the methodology stipulated in the Iranian Standard ISIRI 9044 PB Type P2 with a Janka ball (compatible with the American ASTM D1037-99 standard specifications). However, as the aim of the present project was mainly focused on how sepiolite would affect thermal conductivity and the possible alteration in the curing of resin that would be affected by the addition of sepiolite, hardness was measured at different depths of immersion of the Janka ball. This modification to the standard was made in this project to give a better understanding of how hardness could be altered at different depths, so that any alterations in the treatments studied here can be compared and analyzed. 

The diameter of the ball was 11.28 mm (projected area of 100 mm^2^). The dimensions of the specimens were 75 × 150 mm^2^. Two specimens were bound together to reach the minimum thickness of 25 mm; two penetrations were made on each specimen. A loading test was uniformly applied at a rate of 4 mm/min. Hardness was measured at five consecutive penetration depths of the modified Janka ball; that is, hardness loading was measured at 1, 2, 3, 4, and 5 mm of the penetration of the ball into the OSL specimens. 

### 2.7. Statistical Analysis

SAS software program was used to carry out statistical analysis in the present study (version 9.2; 2010). To discern significant differences among different treatments and produced panels, one-way analysis of variance was performed at a 95% level of confidence. Then, Duncan’s multiple range test (DMRT) was done for the grouping among treatments for each property. In order to find degrees of similarities among different treatments based on the all properties studied here, hierarchical cluster analysis from SPSS/18 (2010) software was used. For graphical statistics (fitted-line, contour, and surface plots), Minitab software was utilized (version 16.2.2; 2010). 

## 3. Results and Discussion

**Figure 5:**
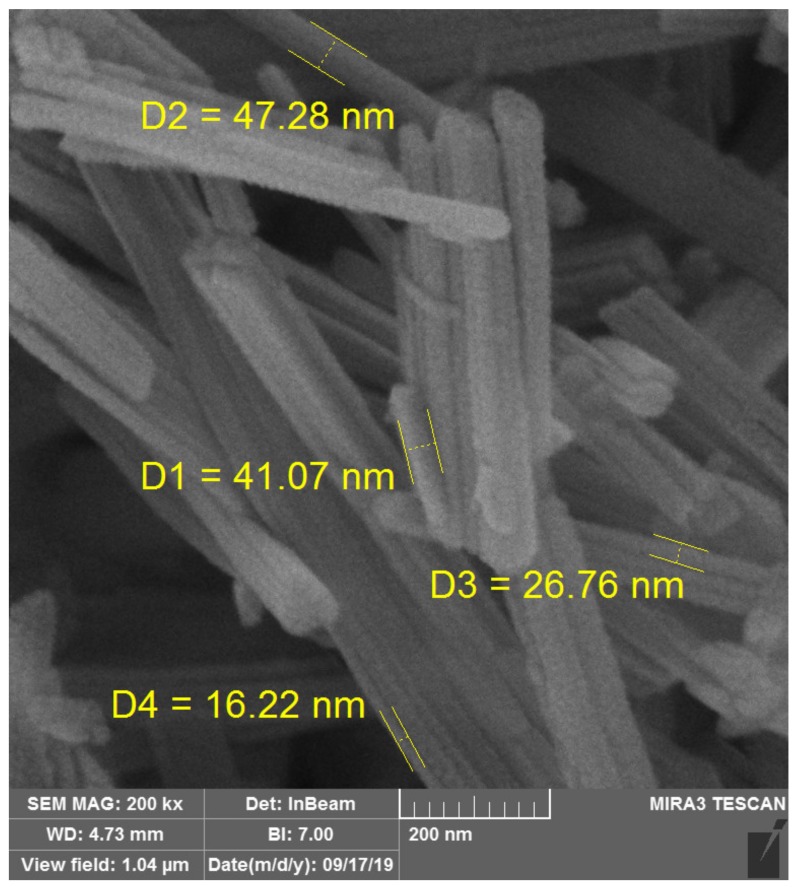
SEM image showing sepiolite nanostrands.

**Figure 6:**
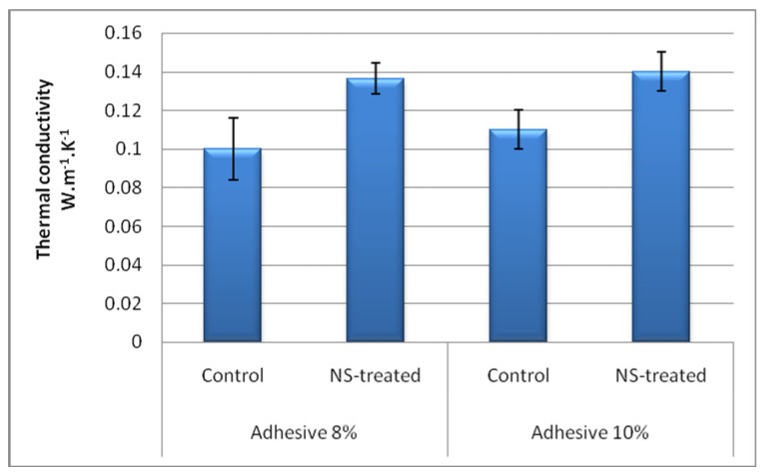
Mean thermal conductivity coefficients (W·m^−1^·K^−1^) for the four treatments (NS = nano-sepiolite).

SEM imaging of sepiolite showed sepiolite nanostrands with a mean aspect ratio of 1:15 (Figure 5). The results of the thermal conductivity coefficient measurement revealed that the highest and lowest values were observed in Adh10-NS and Adh8 panels, respectively (Figure 6). The addition of sepiolite increased thermal conductivity in panels with 8% and 10% adhesive contents by 36% and 40%, respectively. The increase in thermal conductivity was mainly attributed to two basic reasons. Firstly, the addition of sepiolite with a mineral nature and high hardness acted as an effective resin filler. Similarly, the improving effect of other minerals (nano-wollastonite) was previously reported to increase the mechanical performance in composite panels, and also to improve the shear strength of UF resin [41]. The addition of sepiolite was also reported to increase thermal conductivity in polypropylene-based wood–plastic composites, though the thermal conductivity of sepiolite itself is not very high [42]. The thermal conductivity of sepiolite was measured in the present study to be 3.90 ± 0.31 (W·m^−1^·K^−1^) using the same procedure and apparatus. The thermal conductivity of sepiolite is much higher than wood, which is reported to be 0.16 and 0.12 (W·m^−1^·K^−1^) for hardwoods and softwoods, respectively [43]. The thermal stability of polylactide/sepiolite nanocomposites was also improved by introducing sepiolite into the composite [25]. These increases were attributed to sepiolite acting as a cross-linking agent in the composite polymer matrix, ultimately improving the overall integrity of the whole composite. In the present study, the improved integrity in the whole OSL composite eventually resulted in an improvement in thermal conductivity (Figure 6). Similar improvements in integrity and mechanical strengths of composites and resins were previously reported to be achieved by the addition of different minerals at nano- and microscales [36,38,44]. Wollastonite was also reported to improve thermal conductivity in medium-density fiberboard and OSL panels, through which physical and mechanical properties were eventually improved [14,38,44]. Wollastonite also improved the shear strength of polyvinyl acetate resin, as a popular resin in the wood-working industry [41]. The cited authors attributed the improvement to the wollastonite nano-fibers, acting as a hard filler and supporting the whole resin and composite matrix as a strong scaffold. With regard to the fact that the addition of any materials may change the chemical behavior and curing time of a resin, further studies should be carried out to chemically investigate the effects of addition of nano-sepiolite at different content levels on the curing time of UF resin.

Secondly, wood is, in principal, considered a thermal insulator with a very low thermal conductivity; therefore, the addition of a SiO_2_-based material improved the overall thermal conductivity of the whole composite materials. The improved conductivity, in turn, increased the integrity of the whole composite, ultimately facilitating the transfer of heat throughout the OSL-composite mat.

**Figure 7:**
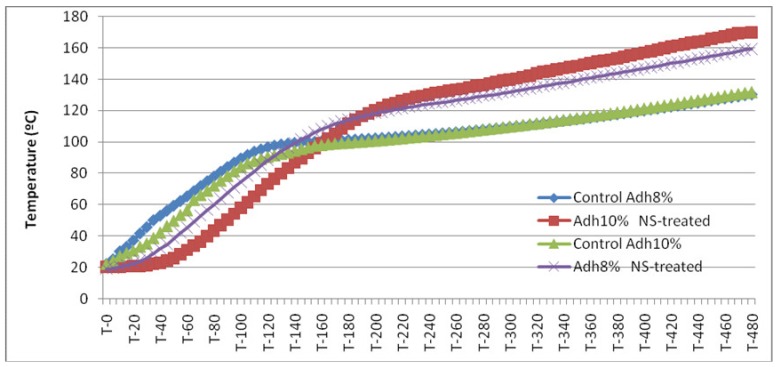
Mean core temperature (°C) at 5 s intervals for the four treatments studied.

The measurement of core temperature in panels showed a general increasing trend in all treatments (Figure 7). Both sepiolite-treated panels showed a significantly higher core temperature for the second half of hot-press time (from 200 s onward). This was attributed to the high thermal conductivity coefficient of sepiolite [36]. Wollastonite, another mineral material with a higher thermal conductivity than wood, was previously reported to have an increasing trend in the core temperature of the MDF mat [37]. Sepiolite-treated panels with 10% of adhesive content had a higher core-temperature in comparison to sepiolite-treated panels, containing 8% adhesive (Figure 7). This was consistent with the higher thermal conductivity of the same panels. For the first 2–3 min, temperature increase was slower in sepiolite-treated panels compared with the control panels, and the increase in core temperature started later. The improved thermal conductivity also facilitated the evaporation of water content in wood strips, a process that, in turn, decreased temperature. It was hypothesized that the evaporation of water took place close to the surface in an earlier stage of heating and/or a larger volume and that evaporation deferred the increase in the temperature of the core. Also, from Figure 7 it can be seen that, for the non-treated material, the temperature reached a plateau at 100 °C, when water began to evaporate. This deviation was less pronounced for the sepiolite-treated material. In these cases, the change into a plateau-like behaviour happened at a higher temperature. It might be that the free water/vapour bounded to sepiolite and higher temperatures were necessary for the phase transition. The improved thermal conductivity also facilitated the evaporation of water content in wood strips, a process that, in turn, decreased temperature. However, as the hot-press continued and from around 200–230 s onward, most of the water content in wood strips and adhesive was evaporated. This was ultimately translated to the significant difference between the core temperatures of sepiolite-treated panels and control panels. 

With regard to the fact that an improved thermal conductivity in a thermo-set resin such as UF would ultimately be translated into a more effective polymerization of the resin, the hardness of the panel was measured at different depths of penetration. The measurement of hardness in the present study was slightly modified in a way that hardness was measured at different depths of penetration of Janka ball. This modification to the standard procedure was done to find out how the improved conductivity could affect hardness at different layers and depths of penetration of the ball. A comparison of values at similar depths of immersion of Janka ball would provide a better understanding of the effects of the improved thermal conductivity on hardness, as a mechanical property. 

**Figure 8:**
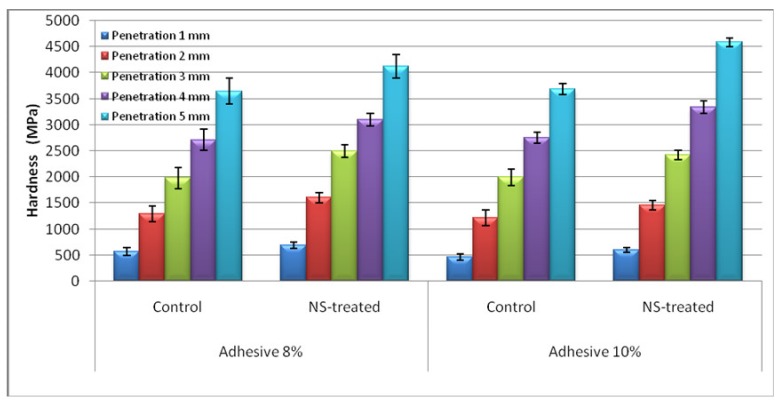
Hardness values (MPa) for the four treatments studied; for each treatment, five hardness values represent for five depths of penetration (NS = nano-sepiolite).

Measurement of hardness did not show significant difference between the control panels with 8% and 10% of adhesive contents (Figure 8). In some cases, hardness in Adh8% panels was even higher, though not statistically significant. This indicated that the increase in resin content from 8% to 10% did not have a significant effect on the hardness property. Similar results were reported in OSL panels containing wollastonite [45]. The cited authors expressed compactness as the decisive factor on the mechanical properties of OSL panels, rather than adhesive content. However, the addition of sepiolite significantly increased hardness values in all penetration depths. Hardness slightly increased as sepiolite content increased. Considering the fact that the amount of sepiolite used in panels was very small, and therefore it could not physically have an impact on the hardness increase, the increase in hardness was attributed to the improvement in the thermal conductivity of the panels. This improvement, in turn, may have facilitated the resin-curing process, eventually increasing the hardness. However, further studies should be carried out in future on resin-curing to have a better understanding on how sepiolite chemically affects UF-resin. Chemical studies like resin-curing and FTIR would possibly explain why the increases in hardness values were so low, contrary to some previous studies demonstrating the high impact of low amounts of nanofillers on different properties of resins and adhesives [46,47,48,49]. 

**Figure 9:**
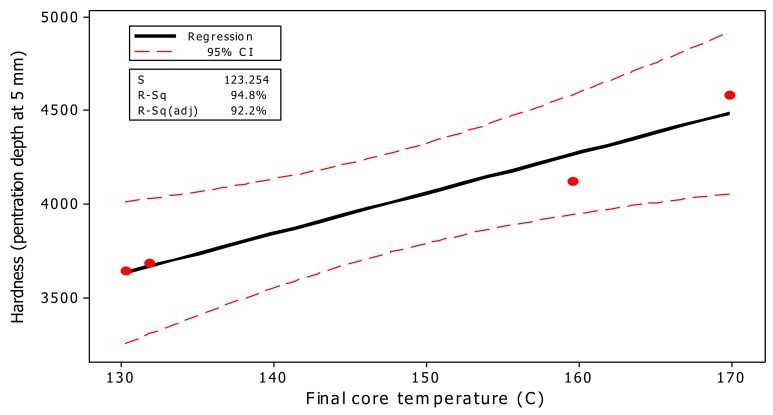
Fitted-line plot between hardness (at 5 mm depth of penetration) versus final core temperature ºC (at 480 s) for the four treatments studied in the present project.

**Figure 10:**
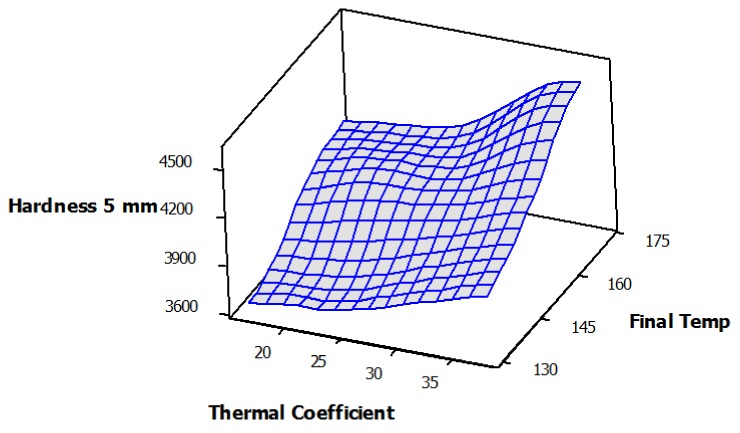

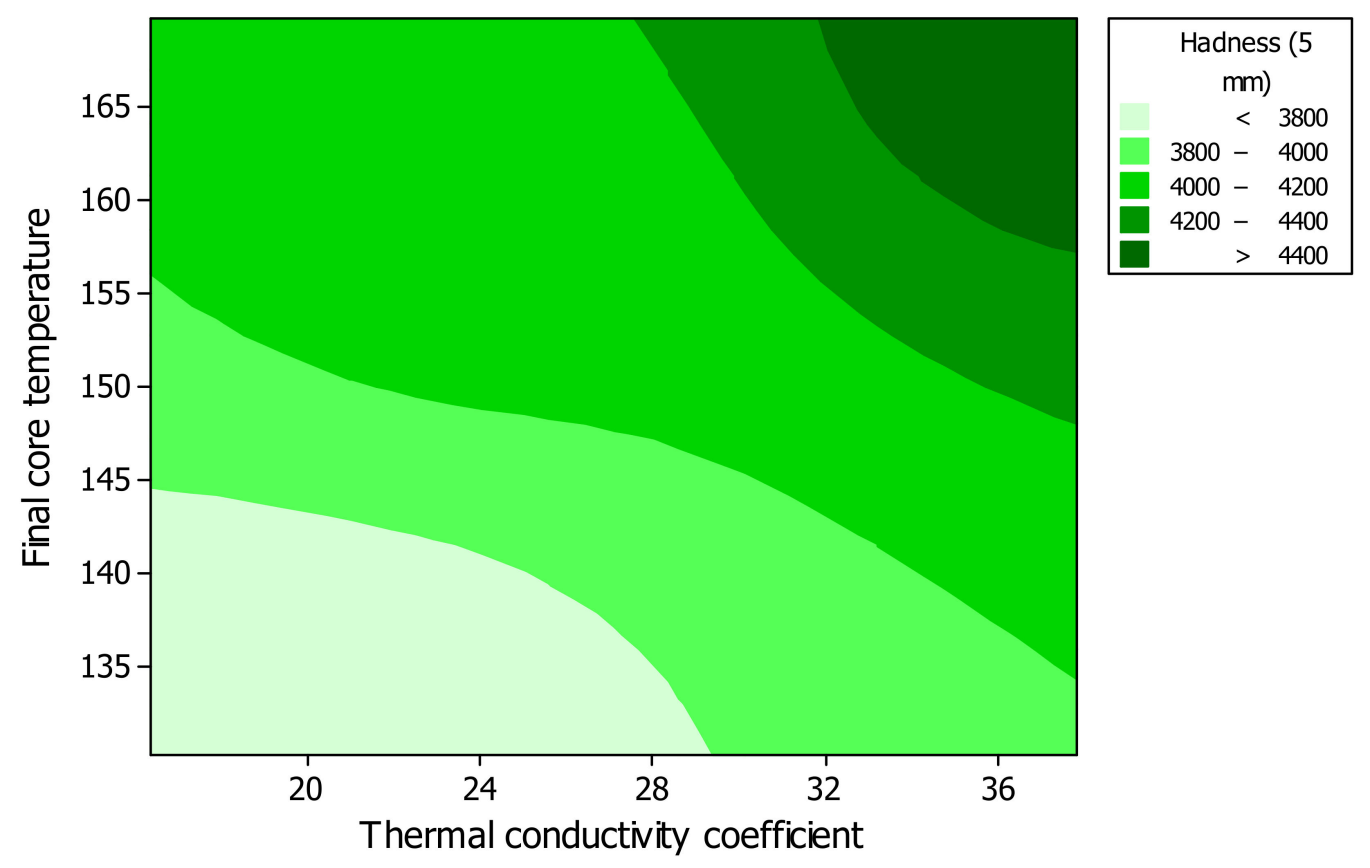
Surface analysis (A) and contour plot (B) among hardness (at 5 mm depth of penetration), versus final core temperature (at 480 s) and thermal conductivity coefficient.

Regression analysis among different properties showed the high and significant R-square values between all of them. The highest regression value (R-square of 94.8%) was observed between the final core temperature of panels after hot-press time (8 min) versus hardness values at a 5-mm depth of penetration (Figure 9). The high regression indicated that as the heat-transfer process into the core of composite mats was enhanced, properties were improved as a result of facilitated resin-curing. Moreover, the surface and contour analysis of the three properties of thermal conductivity coefficient, the final core temperature of mats, and the hardness values clearly demonstrated a smooth mutual direct relationship among all properties (Figure 10). 

**Figure 11:**
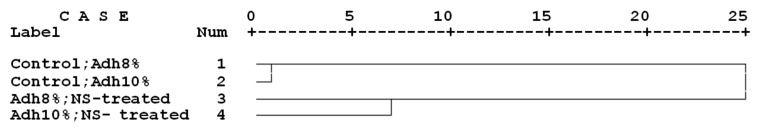
Cluster analysis of the four treatments based on thermal conductivity coefficient, final temperature, and hardness values (Adh = adhesive content; NS = nano-sepiolite).

Cluster analysis of the four treatments based on the thermal conductivity coefficient, final temperature measured at the core of mat during hot-pressing, and hardness values demonstrated a clear distinction between the two control treatments and the sepiolite-treated panels (Figure 11). This indicated high similarity of panels produced with 8% and 10% UF-adhesive. Therefore, it can be concluded that for OSL panels without sepiolite content, 8% adhesive content would be enough to acquire the same hardness and thermal conductivity as can be acquired when 10% adhesive is used. Sepiolite-treated panels with 8% and 10% adhesive contents showed a slight difference with each other. This indicated that these two treatments have significant differences with regard to their hardness and conductivity properties. Therefore, panels with a higher adhesive content of 10% would be recommended for the end-users for whom hardness is of vital importance. However, for end-users to whom insulation of coldness and warmth is important, panels with lower adhesive and sepiolite contents are more recommended.

## 4. Conclusions

Oriented strand lumber is an engineered wood-based material that has been studied rather vastly in recent past decades. The present project was primarily carried out to examine the potential impact of sepiolite at nanoscale on the thermal conductivity coefficient of OSL panels. Sepiolite was mixed with UF resin for 20 min prior to being sprayed onto wood strips in a rotary drum. Ten percent of sepiolite was mixed with the resin. Hardness values at five penetration depths were measured to check the effect of improved thermal conductivity on at least one mechanical property. The measurement of core temperature in OSL panels revealed that sepiolite-treated panels with 10% resin content had a higher core temperature in comparison to the ones containing 8% resin. Results showed significant increases in the thermal conductivity coefficient of sepiolite-treated panels. The increased thermal conductivity was translated into the facilitated heat transfer to the core section of mats, ultimately increasing hardness values. Considering the fact that the amount of sepiolite used in panels was very low, and therefore it could not physically have an impact on the hardness increase, the significant increase in hardness was attributed to the improvement in thermal conductivity and consequent more complete curing of resin. However, further specific studies on resin curing should be carried out to clarify why the impact of sepiolite on hardness values is not comparable to other nanofillers. A significant positive direct relationship was found between accelerated heat-transfer versus hardness values at higher penetration depths. As the results indicated that adhesive contents of 8% and 10% did not have a significant positive effect on hardness in control OSL panels (panels with no sepiolite content), a lower adhesive content of 8% is recommended for the industry to save resin and offer a more competitive price. However, in sepiolite-treated panels, a higher sepiolite content is recommended to achieve maximum hardness values. 

## Figures and Tables

**Table 1:** XRF analysis of sepiolite from Tanbo region in Iran.

Sepiolite compounds	Content by mass (wt. %)
SiO_2_	48.4
Al_2_O_3_	1.3
Fe_2_O_3_	5.9
MgO	15.4
SO_3_	0.6
CaO	8.0
Na_2_O	0
K_2_O	0.2
MnO	0.2
SrO	0.1
ZnO	0.2
BaO	0.5
L.O.I.*	18.88
Total (wt. %)	99.68

*Loss on Ignition.

## References

[1] Zhang C., Smith G.D. (2010). Effects of nanoclay addition to phenol-formaldehyde resin on the permeability of oriented strand lumber. Wood Fiber Sci..

[2] Akrami A. (2010). Development and characterization of oriented strand boards made from the European hardwood species: Beech (*Fagus sylvatica* L.) and poplar (*Populus tremula* L.). Ph.D. Thesis.

[3] Candan Z., Akbulut T. (2014). Nano-engineered plywood panels: Performance properties. Compos. Part B.

[4] Malanit P., Kyokong B., Laemsak N. (2005). Oriented strand lumber from rubberwood residues. Walailak J. Sci. Technol..

[5] Papadopoulos A.N. (2010). Chemical modification of solid wood and wood raw materials for composites production with linear chain carboxylic acid anhydrides: A brief Review. BioResources.

[6] Kelly M.W. (1977). Critical Literature Review of Relationships between Processing Parameters and Physical Properties of Particleboard.

[7] Maloney T.M. (1993). Modern Particleboard and Dry Process Fibreboard Manufacturing.

[8] Walker J.C.F. (2006). Primary Wood Processing: Principles and Practice.

[9] Papadopoulos A.N., Bikiaris D.N., Mitropoulos A.C., Kyzas G.Z. (2019). Nanomaterials and chemical modification technologies for enhanced wood properties: A review. Nanomaterials.

[10] Bayani S., Taghiyari H.R., Papadopoulos A.N. (2019). Physical and mechanical properties of thermally-modified beech wood impregnated with silver nano-suspension and their relationship with the crystallinity of cellulose. Polymers.

[11] Taghiyari H., Esmailpour A., Papadopoulos A. (2019). Paint Pull-Off Strength and Permeability in Nanosilver-Impregnated and Heat-Treated Beech Wood. Coatings.

[12] Hassani V., Taghiyari H.R., Schmidt O., Maleki S., Papadopoulos A.N. (2019). Mechanical and physical properties of Oriented Strand Lumber (OSL): The effect of fortification level of nanowollastonite on UF resin. Polymers.

[13] Papadopoulos A.N., Taghiyari H.R. (2019). Innovative wood surface treatments based on nanotechnology. Coatings.

[14] Li D. (2012). Nanostructuring materials towards conventionally unachievable combination of desired properties. J. Nanomater. Mol. Nanotechnol..

[15] Papadopoulos A.N., Avtzis D., Avtzis N. (2003). The biological effectiveness of wood modified with linear chain carboxylic acid anhydrides against the subterranean termites Reticulitermes flavipes. Holz Als Roh-Und Werkstoff.

[16] Palanti S., Feci E., Predieri G., Francesca V. (2012). Copper complexes grafted to amino-functionalized silica gel as wood preservatives against fungal decay: Mini-blocks and standard test. BioResources.

[17] Salari A., Tabarsa T., Khazaeian A., Saraeian A. (2013). Improving some of applied properties of oriented strand board (OSB) made from underutilized low quality Paulownia (*Paulownia fortunei*) wood employing nano-SiO_2_. Ind. Crop. Prod..

[18] Taghiyari H.R. (2014). Nanotechnology in Wood and Wood-Composite Materials. J. Nanomater. Mol. Nanotechnol..

[19] Mantanis G., Papadopoulos A.N. (2010). The sorption of water vapour of wood treated with a nanotechnology compound. Wood Sci. Technol..

[20] Mantanis G.I., Terzi E., Kartal S.N., Papadopoulos A.N. (2014). Evaluation of mold, decay and termite resistance of pine wood treated with zinc and copper based nanocompounds. Int. Biodeterior. Biodegrad..

[21] Ismita N., Lokesh C. (2017). Effects of different nanoclay loadings on the physical and mechanical properties of *Melia composite* particle board. Bois Et For. Des Trop..

[22] Papadopoulos A.N., Duquesnoy P., Cragg S.M., Pitman A.J. (2008). The resistance of wood modified with linear chain carboxylic acid anhydrides to attack by the marine wood borer Limnoria quadripunctata Hothius. Int. Biodegrad. Biodeterior..

[23] Taghiyari H.R., Majidinajafabad R., Vahidzadeh R. (2018). Wollastonite to hinder growth of *Aspergillus niger* fungus on cotton textile. Anais da Academia Brasileira de Ciencias.

[24] Olivato J.B., Marini J., Yamashita F. (2017). Sepiolite as a promising nanoclay for nano-biocomposites based on starch and biodegradable polyester. Mater. Sci. Eng. C.

[25] Liu M., Pu M., Ma H. (2012). Preparation, structure and thermal properties of polylactide/sepiolite nanocomposites with and without organic modifiers. Compos. Sci. Technol..

[26] Tartaglione G., Camino D.T. (2008). Thermal and morphologicalcharacterization of organically modified sepiolite. Microporous Mesoporous Mater..

[27] Xie S.B., Zhang S.M., Wang F.S., Yang M.S., Seguela R., Lefebvre J.M. (2007). Preparation, structure and thermomechanical properties of nylon-6 nanocomposites with lamella-type and fiber type sepiolite. Compos. Sci. Technol..

[28] Chen H.X., Zheng M.S., Sun H.Y., Jia Q.M. (2007). Characterization and properties of sepiolite/polyurethane nanocomposites. Mater. Sci. Eng. A.

[29] Ruiz-Hitzky E. (2001). Molecular access to intracrystalline tunnels of sepiolite. J. Mater. Chem..

[30] Falco G., Giulieri F., Volle N., Pagnotta S., Sbirrazzuoli N., Disdier E.P., Mija A. (2019). Self-organization of sepiolite fibbers in a biobased thermoset. Compos. Sci. Technol..

[31] Duaresne E., Moins S., Alexandre M., Dubois P. (2007). How can nanohybrids enhance polyester sepiolite nanocomposite properties. Macromol. Chem. Phys..

[32] Zheng Y.P., Zheng Y. (2006). Study on sepiolite-reinforced polymeric nanocomposites. J. Appl. Polym. Sci..

[33] Ma J., Bilotti E., Peijs T., Darr J.A. (2007). Preparation of poly- propylene/sepiolite nanocomposites using supercritical CO_2_ assisted mixing. Eur. Polym. J..

[34] Liu Y., Zhao J., Deng C.L., Chen L., Wang D.Y., Wang Y.Z. (2011). Flame-retardant effect of sepiolite on an intumescent flame-retardant polypropylene system. Ind. Eng. Chem. Res..

[35] Li X., Gao Q., Xia C., Li J., Zhou X. (2019). Urea formaldehyde resin resultant plywood with rapid formaldehyde release modified by tunnel-structured sepiolite. Polymers.

[36] Soltani A. (2019). Presentation and Examination of the Occurrence of Sepiolite Mineral in Tanbo Region, Southeastern Minab, Iran. Res. Earth Sci..

[37] Taghiyari H.R., Mobini K., Sarvari Samadi Y., Doosti Z., Karimi F., Asghari M., Jahangiri A., Nouri P. (2013). Effects of nano-wollastonite on thermal conductivity coefficient of medium-density fiberboard. J. Mol. Nanotechnol..

[38] Taghiyari H.R., Moradiyan A., Farazi A. (2013). Effect of nanosilver on the rate of heat transfer to the core of the medium density fiberboard mat. Int. J. Bio-Inorg. Hybrid Nanomater..

[39] Taghiyari H.R., Karimi A., Tahir P.M.D., Choo A.C.Y., Hakeem K.R., Jawaid M., Alothman O.Y. (2015). Effects of Nanotechnology on Fluid Flow. In Agricultural and Wood-Based Composites Materials. Agricultural Biomass Based Potential Materials.

[40] Taghiyari H.R., Norton J., Tajvidi M. (2017). Effects of Nano-Materials on Different Properties of Wood-Composite Materials. Bio-based Wood Adhesives: Preparation, Characterization, and Testing.

[41] Esmailpour A., Taghiyari H.R., Hosseinpourpia R., Adamopoulos S., Zereshki K. (2020). Shear strength of heat-treated solid wood bonded with polyvinyl-acetate reinforced by nanowollastonite. Wood Res..

[42] Özdemir F., Çot A., Alma H. (2018). Effect of sepiolite mineral on thermal properties and thermal conductivity of wood plastic composite materials. Turkish J. For..

[43] Bergman T., Lavine A., Incropera F., DeWitt D. (2011). Fundamentals of Heat and Mass Transfer.

[44] Taghiyari H.R., Ghorbanali M., Tahir P.M.D. (2014). Effects of the improvement in thermal conductivity coefficient by nano-wollastonite on physical and mechanical properties in medium-density fiberboard (MDF). BioResources.

[45] Taghiyari H.R., Hassani V., Maleki S., Eckelman C.A. (2017). Effects of nano-wollastonite on screw withdrawal capacity of oriented strand lumber. J. Nanomater. Mol. Nanotechnol..

[46] Chihi M., Tarfaoui M., Bouraoui C., El Moumen A. (2020). Effect of CNTs additives on the energy balance of carbon/epoxy nanocomposites during dynamic compression test. Polymers.

[47] Dul S., Ecco L.G., Pegoretti A., Fambri L. (2020). Graphene/carbón nanotube hybrid nanocomposites: Effect of compression molding and fused filament fabrication on properties. Polymers.

[48] Kumar V.V., Balaganesan G., Lee J.K.Y., Esmaeely Neisiany R., Surendran S., Ramakrishna S. (2020). A review of recent advances in nanoengineered polymer composites. Polymers.

[49] Velmurugan R., Balaganesan G., Gupta N. (2013). Impact loading on glass/epoxy composite laminates with nano clay. Key Eng. Mater..

